# Effectiveness of an Artificial Intelligence–Enabled Intervention for Detecting Clinical Deterioration

**DOI:** 10.1001/jamainternmed.2024.0084

**Published:** 2024-03-25

**Authors:** Robert J. Gallo, Lisa Shieh, Margaret Smith, Ben J. Marafino, Pascal Geldsetzer, Steven M. Asch, Kenny Shum, Steven Lin, Jerri Westphal, Grace Hong, Ron Chen Li

**Affiliations:** 1 Center for Innovation to Implementation, VA Palo Alto Health Care System, Menlo Park, California; 2 Department of Health Policy, Stanford University, Stanford, California; 3 Department of Medicine, Stanford University, Stanford, California; 4 Stanford Healthcare AI Applied Research Team, Division of Primary Care and Population Health, Stanford, California; 5 Kaiser Permanente Division of Research, Oakland, California; 6 Chan Zuckerberg Biohub Network, San Francisco, California

## Abstract

**Importance:**

Inpatient clinical deterioration is associated with substantial morbidity and mortality but may be easily missed by clinicians. Early warning scores have been developed to alert clinicians to patients at high risk of clinical deterioration, but there is limited evidence for their effectiveness.

**Objective:**

To evaluate the effectiveness of an artificial intelligence deterioration model–enabled intervention to reduce the risk of escalations in care among hospitalized patients using a study design that facilitates stronger causal inference.

**Design, Setting, and Participants:**

This cohort study used a regression discontinuity design that controlled for confounding and was based on Epic Deterioration Index (EDI; Epic Systems Corporation) prediction model scores. Compared with other observational research, the regression discontinuity design facilitates causal analysis. Hospitalized adults were included from 4 general internal medicine units in 1 academic hospital from January 17, 2021, through November 16, 2022.

**Exposure:**

An artificial intelligence deterioration model–enabled intervention, consisting of alerts based on an EDI score threshold with an associated collaborative workflow among nurses and physicians.

**Main Outcomes and Measures:**

The primary outcome was escalations in care, including rapid response team activation, transfer to the intensive care unit, or cardiopulmonary arrest during hospitalization.

**Results:**

During the study, 9938 patients were admitted to 1 of the 4 units, with 963 patients (median [IQR] age, 76.1 [64.2-86.2] years; 498 males [52.3%]) included within the primary regression discontinuity analysis. The median (IQR) Elixhauser Comorbidity Index score in the primary analysis cohort was 10 (0-24). The intervention was associated with a −10.4–percentage point (95% CI, −20.1 to −0.8 percentage points; *P* = .03) absolute risk reduction in the primary outcome for patients at the EDI score threshold. There was no evidence of a discontinuity in measured confounders at the EDI score threshold.

**Conclusions and Relevance:**

Using a regression discontinuity design, this cohort study found that the implementation of an artificial intelligence deterioration model–enabled intervention was associated with a significantly decreased risk of escalations in care among inpatients. These results provide evidence for the effectiveness of this intervention and support its further expansion and testing in other care settings.

## Key Points

**Question:**

Is an artificial intelligence (AI) deterioration model–enabled intervention associated with a decreased risk of escalations in care during hospitalization?

**Findings:**

In this cohort study of 9938 patients hospitalized at a single academic center in 2021 and 2022, exposure to the intervention was associated with a 10.4–percentage point absolute risk reduction in the primary composite outcome of rapid response team activation, transfer to the intensive care unit, or cardiopulmonary arrest during hospitalization.

**Meaning:**

Findings of this study suggest that use of an AI deterioration model–enabled intervention was associated with a decreased risk of escalations in care during hospitalization.

## Teaser

This cohort study examines whether the use of an artificial intelligence–enabled deterioration model is associated with a decrease in the risk of escalations in care in hospitalized patients.

## Introduction

Clinicians often care for many hospitalized patients concurrently and may not recognize early signs preceding a patient’s clinical deterioration. Such deterioration can result in substantial morbidity and mortality.^1^ Automated early warning scores (EWSs) help alert clinicians to impending patient clinical deterioration so that preventive or rescue actions can be taken to avoid adverse outcomes. Rich real-time patient electronic health record (EHR) data (eg, vital signs, diagnoses, laboratory results, nursing flowsheets) can be used to create predictive models that can be integrated into the EHR and clinical workflows. Given the ease of training and integrating these models, these EWSs have been quickly and widely implemented. Hundreds of hospitals use one such tool, the Epic Deterioration Index (EDI; Epic Systems Corporation), a machine learning clinical deterioration prediction model.^2^

Despite widespread adoption of EWSs, few methodologically rigorous studies have evaluated their impact on patient outcomes. A recent systematic review identified only 1 patient-level randomized clinical trial,^3^ with that study finding no significant difference with the use of an EWS in the primary outcome of transfer to the intensive care unit (ICU).^4^ Most studies, including one of the largest studies to date (N = 43 949),^5^ have reported associations or pre-post comparisons that are vulnerable to inherent biases. Although the EDI is perhaps the EWS with the widest adoption and reach, to our knowledge, no study has evaluated the effectiveness of the EDI in any context with sufficient methodological rigor for establishing causality. This lack of evidence is concerning as implementation of these models may not have benefit and could potentially cause harm through unintended consequences, such as alert fatigue and resource waste. Randomized clinical trials, the gold standard for evaluating effectiveness, may be difficult or even impractical for many hospital systems to conduct due to cost, technical difficulties in randomization, or potential ethical concerns.

Regression discontinuity designs (RDDs) are a promising tool to evaluate the causal effects of quality improvement interventions outside of a randomized clinical trial.^6^ These designs use arbitrary thresholds in continuous variables as a measurable factor that determines which patients receive the intervention. This strategy allows the threshold to be used as a pseudorandomization method; patients just on either side of the threshold should be similar in both measured and unmeasured confounders. Noise in measurements can even be thought to randomize patients right at the threshold to one intervention or another. The discontinuity in regression lines fit on either side of the threshold estimates the causal effect of the intervention for patients exactly at the threshold.^7^ This method has been used to study the associations between contrast and kidney function,^8^ a readmissions prevention intervention and readmission rates,^9^ and red blood cell transfusion and organ dysfunction.^10^

In this study we describe the application of an RDD to evaluate a pilot implementation of an artificial intelligence (AI) deterioration model–enabled intervention to reduce the risk of escalations in care for hospitalized patients. The EDI was implemented as a model-focused alerting system with an associated nurse and physician collaborative workflow.

## Methods

### Design and Implementation of the Deterioration Model–Enabled Intervention

The EDI is an ordinal logistic regression model that predicts risk of the composite outcome of rapid response team (RRT) activation, ICU transfer, cardiopulmonary arrest, or death while an inpatient. The EDI scores are calculated every 15 minutes using 31 clinical measures captured in the EHR; scores range from 0.0 to 100.0, with higher scores indicating higher predicted risk. The vendor’s algorithm was locally validated based on a cohort of 6232 patients admitted to Stanford Hospital, a quaternary care academic medical center, to predict escalation of care events 6 to 18 hours in advance to provide the medical team time to take preventive measures. An EDI score of 65.0 was chosen as the threshold at which patients were determined to be at high risk of clinical deterioration, based on a validation study finding that this cutoff maximized precision and recall at 20%.^11^ The Stanford University Institutional Review Board deemed this cohort study exempt from review and the patient informed consent requirement because it was not human participant research.

The model was implemented as part of an intervention involving nursing staff and physicians. Once a patient’s EDI score reached 65.0, an automated alert was sent to the patient’s nurse and physician. The alert included instructions to initiate a collaborative workflow, which involved a structured huddle and checklist to assess for potential reasons for clinical deterioration. The team was further instructed to consider preventive actions, although no specific action was required. The prompts provided to the care team for the huddle are described in the eMethods in Supplement 1. Model validation and workflow implementation have been described in detail previously.^11^

The intervention was deployed sequentially in 4 general medical units at Stanford Hospital. The intervention was deployed in the first unit on January 17, 2021, followed by the second on May 16, 2021, and the third and fourth units on October 3, 2021. We refer to units in which the intervention was implemented at a given time as active units.

### Data Collection

The cohort for this analysis included all adults hospitalized in an active unit from January 17, 2021, through November 16, 2022. Data on EDI scores and alerts were obtained from a hospital operations database. Age, gender, race and ethnicity, comorbidities, and primary admission diagnosis were extracted from a hospital EHR research data warehouse. Race and ethnicity were categorized as Asian, Black, Hispanic, White, or other (including Native American, Pacific Islander, self-reported other, or missing). Information on race and ethnicity was collected as part of summary demographic data. The Elixhauser Comorbidity Index was calculated using *International Statistical Classification of Diseases and Related Health Problems, Tenth Revision* (*ICD-10*) diagnosis codes for the 6 months prior to admission according to the method of Quan et al^12^; scores range from –19 to 89, with higher scores indicating higher predicted risk of inpatient mortality. Admission *ICD-10* codes were grouped into categories as described in the eTable in Supplement 1. Transfers to the ICU were determined by a change in the patient encounter location to an ICU. Activation of the RRT team and cardiopulmonary arrest events were obtained from a registry maintained by the hospital quality team.

### Statistical Analysis

We used an RDD leveraging intervention assignment (ie, the alert sent to the care team) when the EDI score reached 65.0. The discontinuity was treated as deterministic despite some patients with EDI scores less than 65.0 having alerts in the database because the database contained silent alerts that were used by the operations team as test alerts; these silent test alerts were not sent to the care team. Additionally, while patients whose goals of care were comfort only were excluded from receiving the intervention, we were not able to identify which patients had comfort care orders at the time of a given EDI score calculation. Since we were unable to accurately separate out patients with silent alerts or comfort care orders in our data set, we included them in the analysis cohort to avoid selection bias, similar to an intention-to-treat analysis for a randomized trial with crossover. Because the EDI score is dynamic and calculated every 15 minutes, the maximum EDI score during hospitalization was used as the running variable with scores censored at the time of an event (RRT activation, ICU transfer, cardiopulmonary arrest, or inpatient death); each hospitalization contributed 1 maximum EDI score for this analysis. Regression lines were then fit on either side of the EDI score threshold of 65.0 using a local linear approach with triangular kernel weights to estimate the discontinuity in the primary outcome of escalations in care, which was defined a priori as a composite of RRT activation, ICU transfer, and cardiopulmonary arrest. Escalations of care were chosen as the primary outcome as these events were believed to be both meaningful and most preventable with an early warning system. A secondary outcome was the composite outcome of RRT activation, ICU transfer, cardiopulmonary arrest, and inpatient death. The Calonico-Cattaneo-Titiunik (CCT) bandwidth selection procedure was used to identify an optimal bandwidth on either side of the EDI score threshold for all analyses.^13^ For the primary outcome, the optimal bandwidth selected by the CCT procedure was 6.09 points; however, this bandwidth seemed to be biased by an outlier point below the threshold. Therefore, we chose a bandwidth of 7 points of the EDI score threshold as a more conservative estimate for the primary analysis. We performed additional analyses across bandwidths of 1 to 15 points on either side of the threshold to evaluate sensitivity to bandwidth choice.

By design, RDDs minimize the risk of confounding at the threshold as long as certain assumptions hold true.^14^ The first assumption is that the assignment variable is continuous and is not manipulated. The EDI produces a continuous score from 0.0-100.0 as the output of a complex logistic regression involving many variables that are unlikely to be intentionally manipulated to change risk scores. Nonetheless, this assumption was tested visually as well as empirically by the McCrary sorting test for bunching in the number of individuals on either side of the threshold that would indicate manipulation. A second assumption is that potential confounders are balanced at the threshold. The covariate balance assumption was tested by estimating an RDD with age, Elixhauser Comorbidity Index, and timing of maximum EDI score since admission as the outcome of interest. As a placebo test, a regression discontinuity (RD) analysis was also performed using data from patients on the pilot units prior to implementation of the intervention, as well as data from patients admitted to nonpilot units from June 1 to September 30, 2021. Since these scores were calculated silently and not shown to clinicians, a significant difference in outcomes for the placebo test cohort would not be expected.

All analyses used 2-sided hypothesis tests with a significance level of *P* < .05. Analyses were conducted in R, version 4.1.2 (R Project for Statistical Computing). The RDDtools package was used to perform local linear RD analyses.^15^

## Results

**Figure 1.**
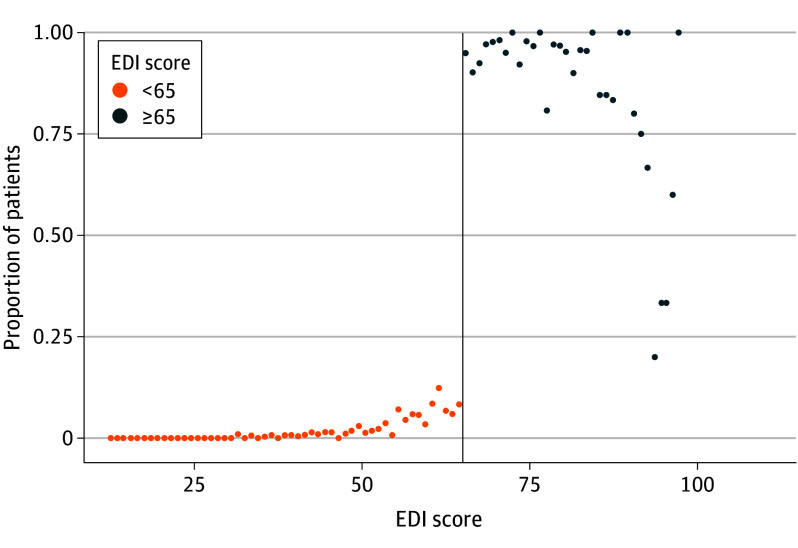
Proportion of Patients Who Triggered Alert Across Epic Deterioration Index (EDI) Scores Patients were binned by 1.0-point increments in the EDI score above and below the threshold of 65.0 (vertical line).

From January 17, 2021, through November 16, 2022, 9938 adult patients were admitted to an active unit. The primary analysis cohort included 963 patients (median [IQR] age, 76.1 [64.2-86.2] years; 455 females [47.7%] and 498 males [52.3%]) whose EDI score was within the bandwidth of 7 points on either side of the threshold of 65.0. The median (IQR) Elixhauser Comorbidity Index score in the primary analysis cohort was 10 (0-24). Table 1 provides information on demographic and clinical characteristics of the entire patient population and of the cohort whose EDI score was within the bandwidth. There was a clear discontinuity in assignment to the intervention observed at the threshold but not at any other EDI scores, as shown in Figure 1. The McCrary sorting test did not show evidence of score manipulation, and eFigure 1 in Supplement 1 shows a histogram of the maximum EDI score distribution.

**Table 1.** Baseline Characteristics of the Total Cohort and According to EDI Score Threshold

Characteristic	No. (%)				
	Within EDI score bandwidth^a^ (n = 953)		Total cohort (N = 9896)		
	Maximum EDI score <65.0 (n = 603)	Maximum EDI score ≥65.0 (n = 350)	Maximum EDI score <65.0 (n = 9125)	Maximum EDI score ≥65.0 (n = 771)	Overall cohort
Age, median (IQR), y	75.3 (62.7-85.3)	77.7 (65.6-87.4)	66.0 (50.8-78.6)	78.7 (66.7-87.4)	67.1 (51.9-79.5)
Gender					
Male	315 (52.2)	183 (52.3)	4782 (52.4)	413 (53.6)	5195 (52.5)
Female	288 (47.8)	167 (47.7)	4342 (47.6)	358 (46.4)	4700 (47.5)
Missing	0	0	1	0	1
Race and ethnicity^b^					
Asian	101 (16.7)	76 (21.7)	1479 (16.2)	163 (21.1)	1642 (16.6)
Black	32 (5.3)	26 (7.4)	614 (6.7)	55 (7.1)	669 (6.8)
Hispanic	105 (17.4)	39 (11.1)	1989 (21.8)	97 (12.6)	2086 (21.1)
White	319 (52.9)	170 (48.6)	4398 (48.2)	376 (48.8)	4774 (48.2)
Other^c^	151 (25.0)	78 (22.3)	2634 (28.9)	177 (23.0)	2811 (28.4)
Comorbidities					
CHF	172 (28.5)	95 (27.1)	1568 (17.2)	214 (27.8)	1782 (18.0)
Kidney disease	170 (28.2)	99 (28.3)	1888 (20.7)	209 (27.1)	2097 (21.2)
Diabetes	176 (29.2)	99 (28.3)	2025 (22.2)	193 (25.0)	2218 (22.4)
Liver disease	113 (18.7)	54 (15.4)	1538 (16.9)	118 (15.3)	1656 (16.7)
CPD	123 (20.4)	71 (20.3)	1418 (15.5)	139 (18.0)	1557 (15.7)
Malignancy	122 (20.2)	66 (18.9)	1641 (18.0)	155 (20.1)	1796 (18.1)
Elixhauser Comorbidity Index, median (IQR)	10 (0-24)	11 (0-24)	6 (0-18)	11 (0-25)	6 (0-18)
Length of stay, median (IQR), d	6.4 (3.8-11.6)	8.2 (4.3-14.9)	3.8 (2.1-6.9)	9.2 (5.0-17.4)	4.0 (2.2-7.3)
Admission diagnosis categories^d^					
Information available	589	336	8817	741	9558
Infection	96 (16.3)	67 (19.9)	1169 (13.3)	162 (21.9)	1331 (13.9)
Gastrointestinal	58 (9.8)	19 (5.7)	1210 (13.7)	34 (4.6)	1244 (13.0)
Cardiovascular	76 (12.9)	52 (15.5)	1188 (13.5)	88 (11.9)	1276 (13.4)
Respiratory	81 (13.8)	58 (17.3)	608 (6.9)	114 (15.4)	722 (7.6)
Malignancy	19 (3.2)	11 (3.2)	339 (3.8)	34 (4.6)	373 (3.9)

Abbreviations: CHF, congestive heart failure; CPD, chronic pulmonary disease; EDI, Epic Deterioration Index.

^a^ Based on an EDI score within 7 points above or 7 points below 65.0.

^b^ Numbers may not sum to 100% because race and ethnicity are separate concepts in the database. Information on age and comorbidities was missing for 42 patients in our database (10 patients within the 7-point EDI score bandwidth and 32 patients outside the 7-point EDI score bandwidth).

^c^ Other race and ethnicity includes Native American, Pacific Islander, self-reported other, or missing.

^d^ Defined as described in the eTable in Supplement 1.

**Figure 2.**
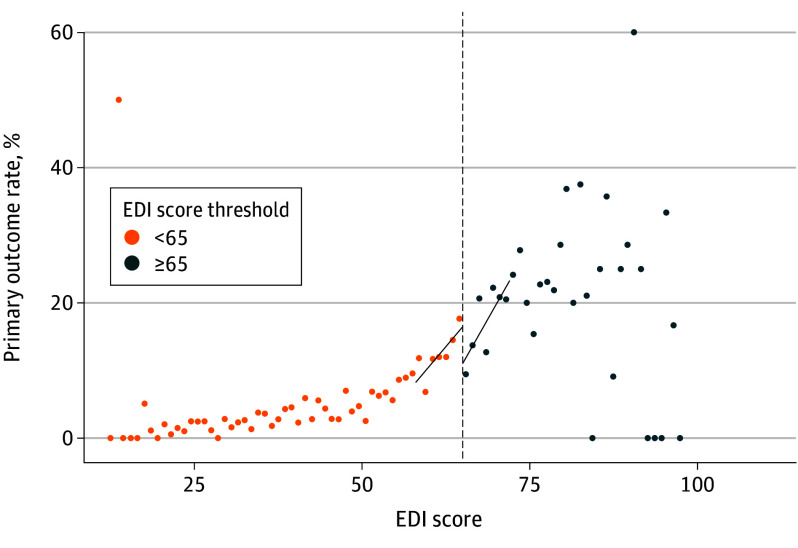
Rate of Primary Outcome Across Epic Deterioration Index (EDI) Scores Patients were binned by 1.0-point increments in the EDI score above and below the threshold of 65.0 (vertical line). The primary outcome was activation of the rapid response team, transfer to an intensive care unit, or cardiopulmonary arrest. Linear regression lines fit to the binned primary outcome rate above and below the EDI score threshold are shown.

Rates of the primary outcome (RRT activation, ICU transfer, or cardiopulmonary arrest) by EDI score are shown in Figure 2. The RDD analysis estimated an absolute risk reduction of −10.4 percentage points (95% CI, −20.1 to −0.8 percentage points; *P* = .03) in the primary outcome for patients at the EDI score threshold. eFigure 2 in Supplement 1 shows the results of sensitivity testing across different bandwidth choices, with 95% CIs including 0 at bandwidths greater than 9 points on either side of the threshold. The RD estimate for the secondary outcome (RRT activation, ICU transfer, cardiopulmonary arrest, or inpatient death) was found to be an absolute risk reduction of −7.0 percentage points (95% CI, −16.6 to 2.6 percentage points; *P* = .15) for patients at the EDI score threshold, with event rates by EDI score shown in eFigure 3 in Supplement 1. eFigure 4 in Supplement 1 shows the sensitivity of the secondary outcome to bandwidth choice, with 95% CIs rejecting the null hypothesis at bandwidths of less than 5 points on either side of the threshold. Results of RD analyses for individual component outcomes are shown in Table 2. Rates of documentation for completing the structured huddle are shown in eFigure 5 in Supplement 1.

**Table 2.** Regression Discontinuity Estimates of Primary and Secondary Outcomes^a^

Outcome	Bandwidth^b^	Effect estimate, percentage points (95% CI)	*P* value
Determination of primary outcome^c^			
Manual bandwidth specification	7.00	−10.4 (−20.1 to −0.8)	.03
CCT bandwidth specification	6.09	−11.9 (−22.3 to −1.5)	.03
Local quadratic regression	10.22	−14.3 (−26.0 to −2.6)	.02
Secondary outcome^d^	8.17	−7.0 (−16.6 to 2.6)	.15
Inpatient death	9.72	−0.4 (−6.4 to 5.6)	.88
Transfer to ICU	6.04	−7.5 (−16.7 to 1.6)	.11
RRT activation or cardiopulmonary arrest	8.24	−4.1 (−11.3 to 3.1)	.26

Abbreviations: CCT, Calonico-Cattaneo-Titiunik; ICU, intensive care unit; RRT, rapid response team.

^a^ Effect estimates were calculated using local linear regression with a bandwidth defined by the CCT bandwidth procedure^13^ unless otherwise noted.

^b^ Based on an EDI score within the stated number of points above or below 65.0.

^c^ Primary outcome defined as RRT activation, ICU transfer, or cardiopulmonary arrest.

^d^ Secondary outcome defined as primary outcome or inpatient death.

There was no evidence of a discontinuity for the potential confounders of age (RD estimate, 1.5 years [95% CI, −2.4 to 5.3 years]; *P* = .45), Elixhauser Comorbidity Index (RD estimate, 0.01 points [95% CI, −2.8 to 2.8 points]; *P* = .99), or timing of maximum EDI score since admission (RD estimate, 1.2 days [95% CI, −0.7 to 3.1 days]; *P* = .20) at the EDI score threshold, supporting the underlying assumption of covariate balance at the threshold (eFigure 6 in Supplement 1). eFigure 7 in Supplement 1 shows boxplots of time differences between a first EDI score greater than or equal to 65.0 and the maximum score for the intervention group, with minimal to no lag within the bandwidth of 7 points on either side of the EDI score threshold. As a placebo test for a spurious association between the EDI score and the observed improved outcomes, a similar RD analysis was performed prior to the implementation of the pathway (eFigure 8 in Supplement 1). The CCT bandwidth selector produced a bandwidth of 10.9 points on either side of the EDI score threshold, which included 1731 patients within the bandwidth. That analysis did not find a statistically significant difference at the EDI score threshold (RD estimate, 2.7 percentage points [95% CI, −3.7 to 9.1 percentage points]; *P* = .41).

## Discussion

This cohort study found a significant reduction in rates of escalations of care associated with an AI deterioration model–enabled intervention among hospitalized patients at an academic medical center. While this was a retrospective cohort study, the RD analysis supports the causal interpretation of the observed treatment effect of the intervention. Multiple checks of the validity of the RDD appeared consistent with the necessary assumptions of this method.^7,14^ In sensitivity analyses, we found estimates of improved outcomes across all potential bandwidth choices; however, 95% CIs began to include 0 at larger bandwidths from the EDI threshold of 65.0. While implementation of the intervention was associated with a reduction in escalations of care, we did not find evidence of a statistically significant reduction in inpatient mortality. Overall, our findings provide much-needed evidence for the effectiveness of this intervention and support further expansion and testing in other care settings.

It is important to note that this study does not measure the effectiveness of the EDI score in isolation; rather, we evaluated an intervention package consisting of the model, an alerting mechanism, and a collaborative workflow shared by physicians and nurses.^11^ While no specific clinical actions were required, the medical team was encouraged to leverage what was discussed during the workflow to inform the patient’s care plan and to make contingency plans in case of further clinical deterioration. Our analysis does not attempt to identify and qualitatively assess which components of this workflow led to improved outcomes, only that the intervention as a whole was associated with improved outcomes.

### Limitations

While the RDD minimizes the risk of unmeasured confounding compared with traditional retrospective cohort studies, this study has limitations. We chose to use the maximum EDI score as the assignment variable for feasibility reasons, which may have reduced the statistical power of the study since the EDI is a dynamic model. This choice could result in bias if the intervention significantly affects the value of subsequent EDI scores such that the distribution of potential outcomes no longer varies smoothly across the EDI score threshold. However, we did not see signs of significant delays between a first EDI score greater than or equal to 65.0 and the maximum EDI score. Additionally, we did not see signs of bunching of EDI scores at the threshold, which would suggest the intervention lowered the maximum EDI score. However, if the intervention did significantly lower the maximum EDI score observed for patients above the threshold, we would expect this to bias our results toward the null hypothesis. Additionally, some patients whose goals of care were comfort only and therefore were not eligible to receive the intervention are nonetheless included in this analysis due to difficulty isolating these patients, although again, we would also expect their inclusion to bias our results toward the null hypothesis.

Another potential limitation of this study, similar to any RDD analysis, is the generalizability of the findings. Because we estimated the discontinuity in outcomes specifically for patients whose EDI score was near the threshold score of 65.0, these results may not apply to patients whose EDI score is far above or far below the threshold. For instance, patients with higher EDI scores may be more easily recognizable as deteriorating by the medical team, so any alert-based system may be redundant for them; conversely, patients with lower EDI scores may be at such low risk for deterioration that there is little opportunity for the intervention to change outcomes. These hypotheses could be further evaluated using an RDD by iteratively changing the EDI score threshold used for alerting the medical team. Additionally, these results may not generalize to other hospitals with different patient populations and resources.

Interventions focused on emerging AI technologies are often implemented in care delivery without rigorous evaluations for effectiveness, typically relying on simple pre-post study designs that suffer from confounding and other design flaws that limit internal and external validity. Nevertheless, it is often difficult or impractical to use gold standard study designs, such as randomized clinical trials, in care delivery settings. Our study shows a potential alternative: RDDs, an underused quasi-experimental analysis method that can be applied to assess the effectiveness of a technology-focused intervention using routinely collected data. The RDD method requires few assumptions for inferences to be valid, unlike other methods, such as propensity scores and difference-in-differences.^14^ This analysis method can be used by health systems that may not have the infrastructure or capacity to assess effectiveness in a randomized clinical trial of their own implementation of an early warning score.

## Conclusions

Using an RDD, this cohort study found that implementation of an AI deterioration model–enabled intervention was associated with a significantly decreased risk of escalations in care for patients hospitalized at an academic medical center. Amid the limited evidence base for early warning scores despite widespread adoption, this study provides evidence for their effectiveness and supports further testing of these interventions in other care settings.

## Notes

Supplement 1.
eMethods.
**eFigure 1.** Count of Patients Across Maximum EDI Scores **eFigure 2.** Sensitivity of the Primary Outcome to Bandwidth Choice **eFigure 3.** Rates of the Secondary Outcome Across EDI Scores **eFigure 4.** Sensitivity of the Secondary Outcome to Bandwidth Choice **eFigure 5.** Documentation of Huddle by Score **eFigure 6.** Age (A), Elixhauser Comorbidity Index (B), and Time of Maximum EDI Score Since Admission (C) by EDI Score **eFigure 7.** Time from First EDI Score ≥65.0 to Maximum EDI Score **eFigure 8.** Placebo Test for the Primary Outcome **eTable.** Admission Diagnosis Categories

Supplement 2.
Data Sharing Statement
