# Supplementary material for: Effectiveness of an Artificial Intelligence–Enabled Intervention for Detecting Clinical Deterioration
Source: JAMA Intern Med. 2024 Mar 25;184(5):557–62. doi: 10.1001/jamainternmed.2024.0084 (PMC10964159; doi:10.1001/jamainternmed.2024.0084)
Supplement: Supplement 1. — eMethods. eFigure 1. Count of Patients Across Maximum EDI Scores eFigure 2. Sensitivity of the Primary Outcome to Bandwidth Choice eFigure 3. Rates of the Secondary Outcome Across EDI Scores eFigure 4. Sensitivity of the Secondary Outcome to Bandwidth Choice eFigure 5. Documentation of Huddle by Score eFigure 6. Age (A), Elixhauser Comorbidity Index (B), and Time of Maximum EDI Score Since Admission (C) by EDI Score eFigure 7. Time from First EDI Score ≥65.0 to Maximum EDI Score eFigure 8. Placebo Test for the Primary Outcome eTable. Admission Diagnosis Categories [file jamainternmed-e240084-s001.pdf]

Supplemental Online Content

Gallo RJ, Shieh L, Smith M, et al. Effectiveness of an artificial intelligence–enabled intervention for detecting clinical deterioration. *JAMA Intern Med*. Published online March 25, 2024. doi:10.1001/jamainternmed.2024.0084

eMethods

eFigure 1. Count of Patients Across Maximum EDI Scores

eFigure 2. Sensitivity of the Primary Outcome to Bandwidth Choice

eFigure 3. Rates of the Secondary Outcome Across EDI Scores

eFigure 4. Sensitivity of the Secondary Outcome to Bandwidth Choice

eFigure 5. Documentation of Huddle by Score

eFigure 6. Age (A), Elixhauser Comorbidity Index (B), and Time of Maximum EDI Score Since Admission (C) by EDI Score

eFigure 7. Time from First EDI Score ≥ 65.0 to Maximum EDI Score

eFigure 8. Placebo Test for the Primary Outcome

eTable. Admission Diagnosis Categories

This supplemental material has been provided by the authors to give readers additional information about their work.

©2024 American Medical Association. All rights reserved.

eMethods

Description of the Intervention:
Once a patient's risk score reached 65.0, an alert was sent to the provider and nurse taking care of the patient through the application used by the hospital for clinical communications. Additionally, a best practice alert appeared within the patient's electronic chart, which included prompts to complete a structured huddle. The provider and nurse were instructed to discuss possible reasons for deterioration, with examples given of shock, arrhythmia, aspiration, mental status changes, respiratory failure, and/or other. The team was then prompted to consider specific interventions to prevent decompensation with the following examples: assess aspiration risk/swallow evaluation, new orders, goals-of-care discussion, family meeting, new consult, ICU provider consult, critical care response nurse consult, and/or other.

©2024 American Medical Association. All rights reserved.

eFigure 1. Count of Patients Across Maximum EDI Scores

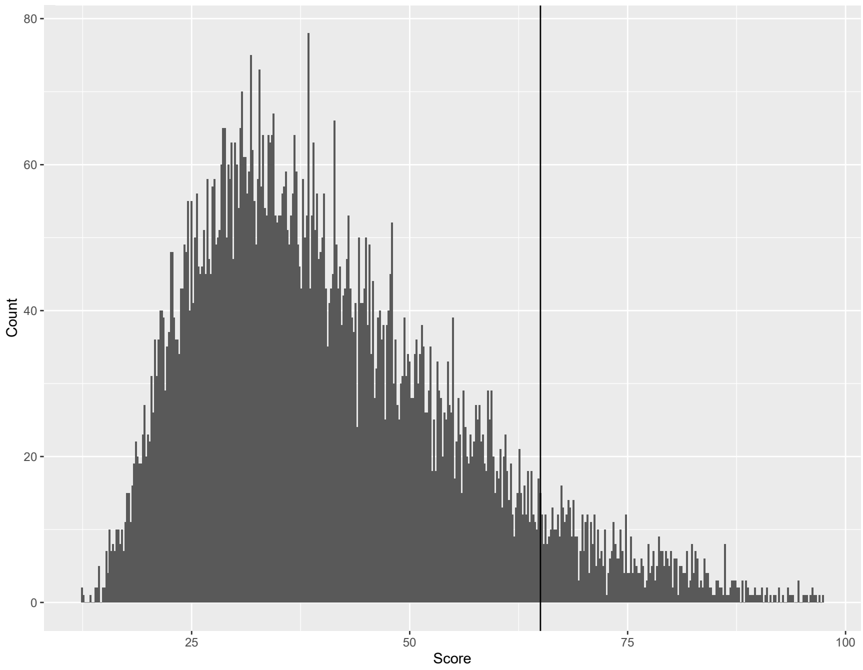

Scores are binned by 0.2 points.

eFigure 2. Sensitivity of the Primary Outcome to Bandwidth Choice

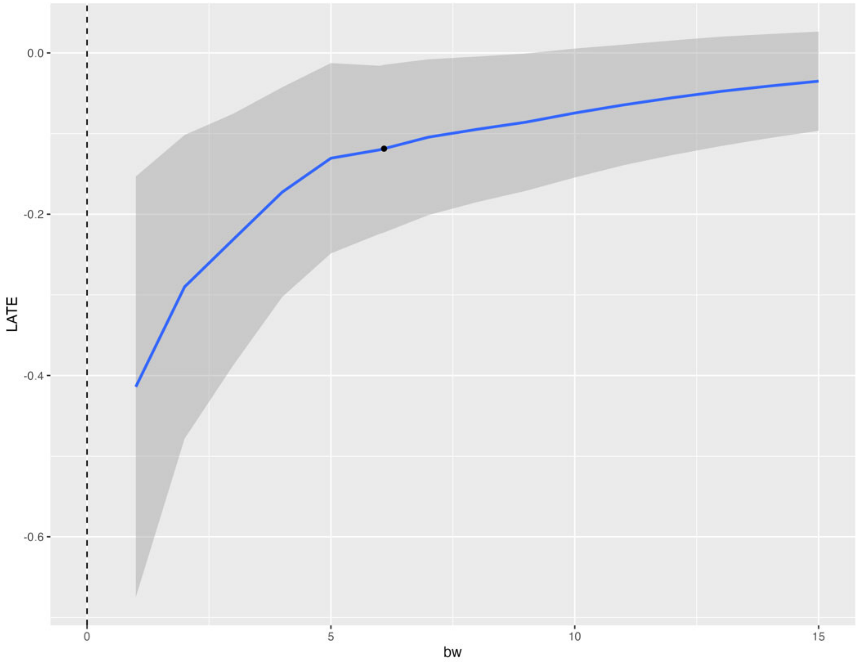

LATE: Local Average Treatment Effect; bw: bandwidth around threshold.
Blue line represents treatment effect estimate (LATE) at a given bandwidth, with gray shaded bounds indicating 95% confidence intervals. Black circle represents the CCT selected bandwidth of 6.09. The treatment effect estimate at that bandwidth was -11.9% (95% CI -22.3 to -1.5; p=0.03).

©2024 American Medical Association. All rights reserved.

eFigure 3. Rates of the Secondary Outcome Across EDI Scores

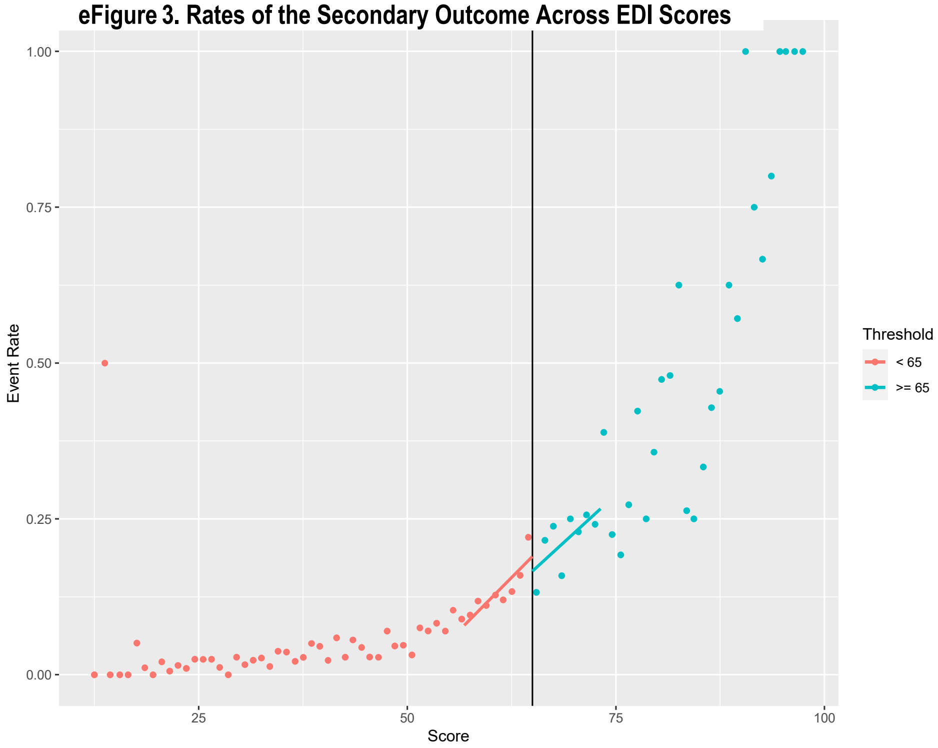

eFigure 4: Sensitivity of the Secondary Outcome to Bandwidth Choice

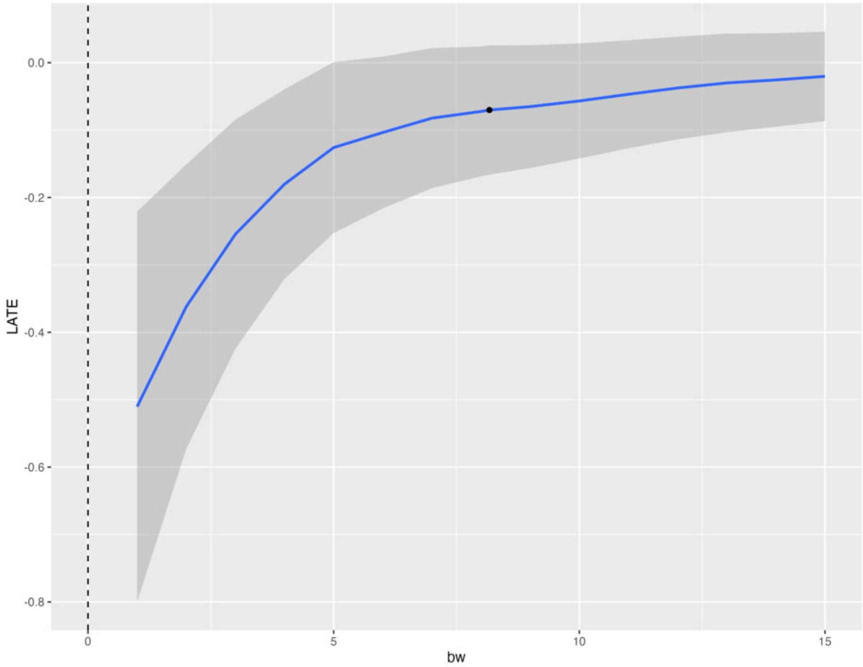

LATE: Local Average Treatment Effect; bw: bandwidth around threshold.
Blue line represents treatment effect estimate (LATE) at a given bandwidth, with gray shaded bounds indicating 95% confidence intervals. Black circle represents the CCT selected bandwidth of 8.17.

©2024 American Medical Association. All rights reserved.

eFigure 5: Documentation of Huddle by Score

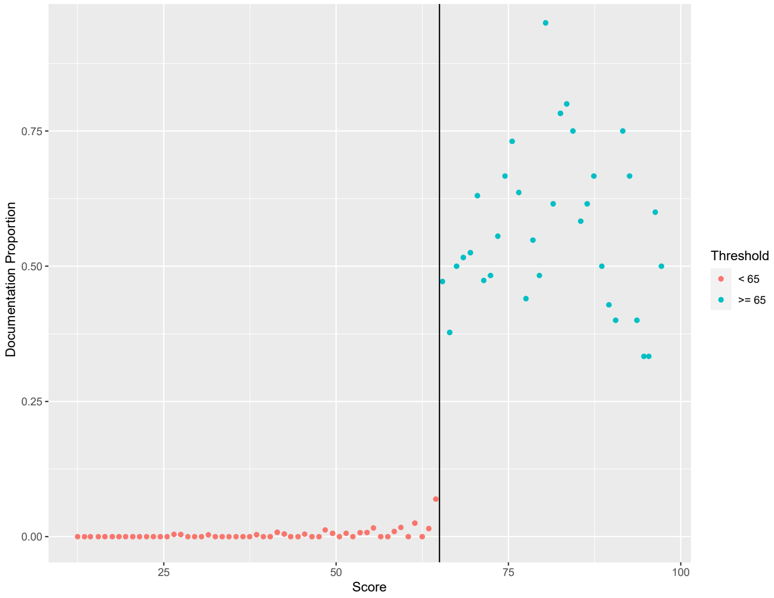

eFigure 6: Age (A), Elixhauser Comorbidity Index (B), and Time of Maximum EDI Score Since Admission (C) by EDI Score

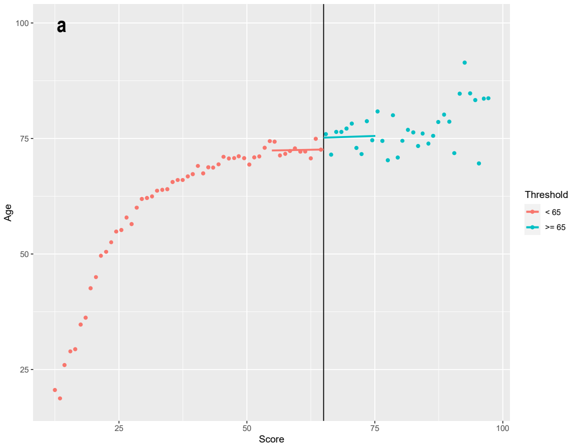

RD estimate 1.5 years; 95% CI -2.4 to 5.3, p=0.45

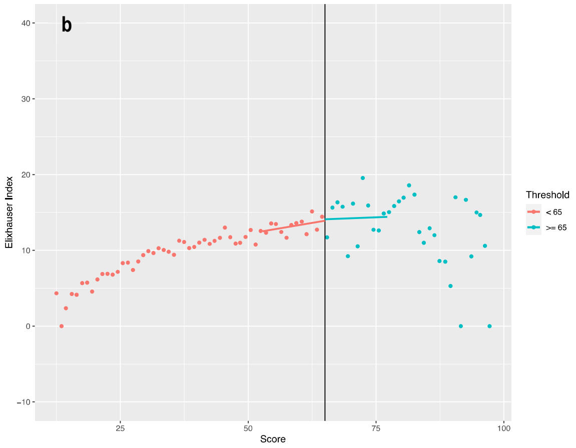

RD estimate 0.01 points; 95% CI -2.8 to 2.8, p=0.99

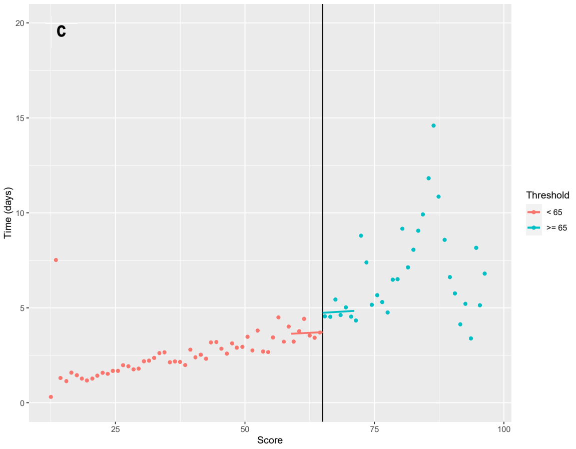

RD estimate 1.2 days; 95% CI -0.7 to 3.1, p=0.20

©2024 American Medical Association. All rights reserved.

eFigure 7: Time from First EDI Score ≥ 65.0 to Maximum EDI Score

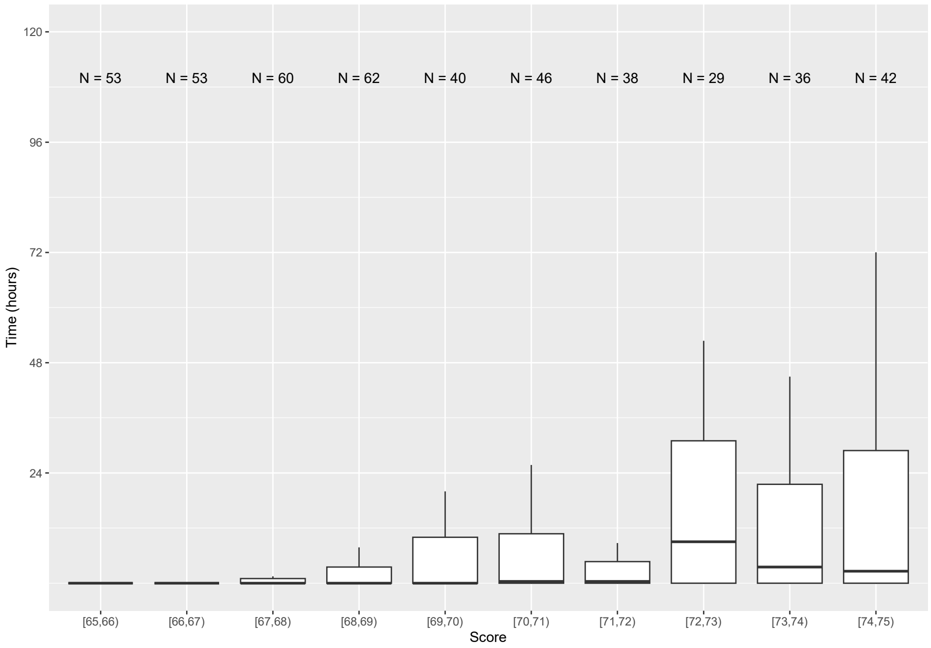

Boxplot showing time from first score ≥ 65 to maximum risk score by maximum risk score. Bold horizontal line represents the median, edges of the box represent interquartile range (IQR), and vertical lines represent the furthest data point within 1.5*IQR from the edges of the box.

eFigure 8: Placebo Test for the Primary Outcome

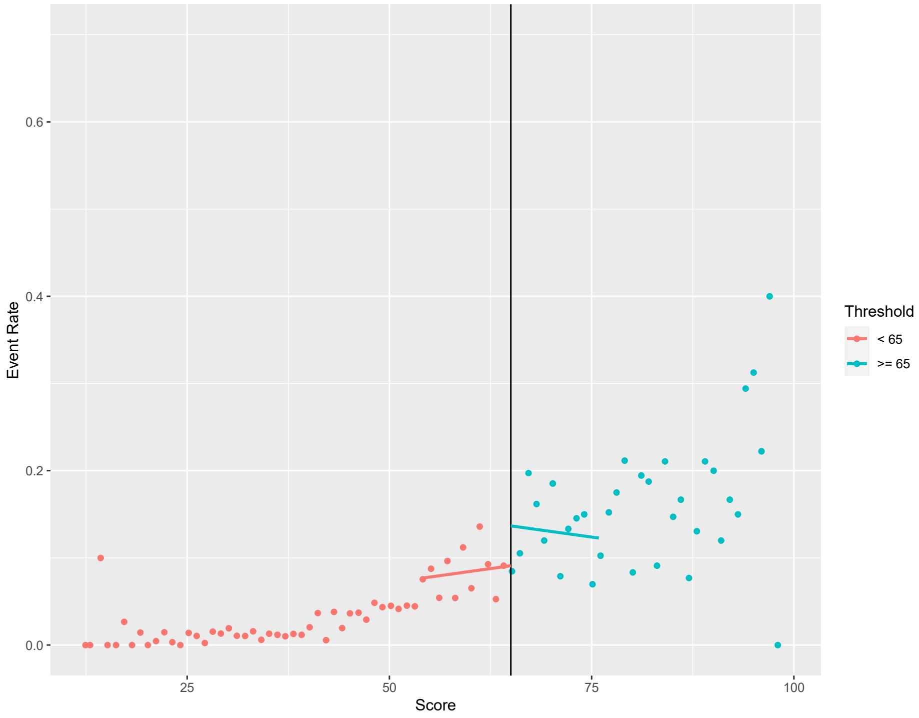

©2024 American Medical Association. All rights reserved.

eTable 1. Admission Diagnosis Categories

Diagnosis Category	ICD-10 Codes
Infection	A400, A401, A403, A408, A409, A4101, A4102, A411, A412, A413, A414, A4150, A4151, A4152, A4153, A4159, A4181, A4189, A419, R509, R7881, J1282, U071, U099, A0225, N10, N110, N111, N118, N119, N12, N136, N151, N3000, N3001, N3010, N3011, N3020, N3021, N3030, N3031, N3080, N3081, N3090, N3091, N340, N341, N342, N343, N390, T83510A, T83511A, T83512A, T83518A, T8351XA, T83592A, T83593A, T83598A, T8359XA, J13, J14, J150, J151, J1520, J15211, J15212, J1529, J153, J154, J155, J156, J157, J158, J159, J160, J168, J17, J180, J181, J188, J189, A46, L0390, L0291, L03011, L03012, L03019, L03031, L03032, L03039, L03111, L03112, L03113, L03114, L03115, L03116, L03119, L03211, L03213, L03221, L03311, L03312, L03313, L03314, L03315, L03316, L03317, L03319, L03811, L03818, L0889, L089, M869
Gastrointestinal	G43D0, G43D1, R100, R1010, R1011, R1012, R1013, R102, R1030, R1031, R1032, R1033, R10811, R10812, R10813, R10814, R10815, R10816, R10817, R10819, R10821, R10822, R10823, R10824, R10825, R10826, R10827, R10829, R1083, R1084, R109, R12, R130, R140, R141, R142, R143, R150, R151, R152, R159, R160, R161, R162, R17, R180, R188, R1900, R1901, R1902, R1903, R1904, R1905, R1906, R1907, R1909, R1911, R1912, R1915, R192, R1930, R1931, R1932, R1933, R1934, R1935, R1936, R1937, R194, R195, R196, R197, R198, I8501, I8511, K2081, K2091, K2101, K2211, K250, K252, K254, K256, K260, K262, K264, K266, K270, K272, K274, K276, K280, K282, K284, K286, K2901, K2921, K2931, K2941, K2951, K2961, K2971, K2981, K2991, K31811, K50011, K50111, K50811, K50911, K51011, K51211, K51311, K51411, K51511, K51811, K51911, K5521, K5701, K5711, K5713, K5721, K5731, K5733, K5741, K5751, K5753, K5781, K5791, K5793, K625, K920, K921, K922, K850, K8500, K8501, K8502, K851, K8510, K8511, K8512, K852, K8520, K8521, K8522, K853, K8530, K8531, K8532, K858, K8580, K8581, K8582, K859, K8590, K8591, K8592, K7030, K7031, K7040, K7041, K7200, K7201, K7210, K7211, K7290, K7291, K9182, K7682, K7460, R7401, K3184, R112, K56609, K529
Cardiovascular	R001, R000, I110, I130, I132, I501, I5020, I5021, I5022, I5023, I5030, I5031, I5032, I5033, I5040, I5041, I5042, I5043, I50810, I50811, I50812, I50813, I50814, I5082, I5083, I5084, I5089, I509, I2101, I2102, I2109, I2111, I2119, I2121, I2129, I213, I214, I219, I21A1, I21A9, I220, I221, I222, I228, I229, I200, I202, I240, I249, I25110, I25112, I25700, I25702, I25710, I25712, I25720, I25722, R072, R0789, R079, G9001, R55, I950, I951, I952, I953, I9581, I9589, I959, I470, I471, I472, I4720, I4721, I4729, I479, I480, I481, I4811, I4819, I482, I4820, I4821, I483, I484, I4891, I4892, I491, I492, I493, I4940, I4949, I495, I498, I499, G43601, G43609, G43611, G43619, I6300, I63011, I63012, I63013, I63019, I6302, I63031, I63032, I63033, I63039, I6309, I6310, I63111, I63112, I63113, I63119, I6312, I63131, I63132, I63133, I63139, I6319, I6320, I63211, I63212, I63213, I63219, I6322, I63231, I63232, I63233, I63239, I6329, I6330, I63311, I63312, I63313, I63319, I63321, I63322, I63323, I63329, I63331, I63332, I63333, I63339, I63341, I63342, I63343, I63349, I6339, I6340, I63411, I63412, I63413, I63419, I63421, I63422, I63423, I63429, I63431, I63432, I63433, I63439, I63441, I63442, I63443, I63449, I6349, I6350, I63511, I63512, I63513, I63519, I63521, I63522, I63523, I63529, I63531, I63532, I63533, I63539, I63541, I63542, I63543, I63549, I6359, I636, I638, I6381, I6389, I639, G450, G451, G452, G453, G458, G459, I2602, I2609, I2692, I2693, I2694, I2699, I160, I161, I169, I674
Respiratory	J410, J411, J418, J42, J430, J431, J432, J438, J439, J440, J441, J449, J470, J471, J479, J80, J95821, J95822, J9600, J9601, J9602, J9610, J9611, J9612, J9620, J9621, J9622, J9690, J9691, J9692, R092, J810, R0902, R0609, J690, J691, J698, R0600, J90, R0602, R0603, J4520, J4521, J4522, J4530, J4531, J4532, J4540, J4541, J4542, J4550, J4551, J4552, J45901, J45902, J45909, J45990, J45991, J45998, J8281, J8282, J8283, J8410, J84111, J84112, J84113, J84114, J84115, J84116, J84117, J8417, J84170, J84178, J8489, J849
Malignancy	C00-C96, D00-D49

©2024 American Medical Association. All rights reserved.
