# Supplementary material for: Effectiveness of an Artificial Intelligence–Enabled Intervention for Detecting Clinical Deterioration
Source: JAMA Intern Med. 2024 Mar 25;184(5):557–62. doi: 10.1001/jamainternmed.2024.0084 (PMC10964159; doi:10.1001/jamainternmed.2024.0084)
Supplement: Supplement 2. — Data Sharing Statement [file jamainternmed-e240084-s002.pdf]

Data Sharing Statement

Gallo. Effectiveness of an Artificial Intelligence–Enabled Intervention for Detecting Clinical Deterioration. *JAMA Intern Med*. Published March 25, 2024.
doi:10.1001/jamainternmed.2024.0084

Data
Data available: No

Additional Information
Explanation for why data not available: Data sharing restricted by Stanford University patient privacy policy.
